# An Atypical Presentation of a Non-neural Granular Cell Tumor

**DOI:** 10.7759/cureus.105836

**Published:** 2026-03-25

**Authors:** Taylor C Boggess, Raafat Makary, Rui Fernandes, Noor Marji

**Affiliations:** 1 Pathology and Laboratory Medicine, University of Florida College of Medicine – Jacksonville, Jacksonville, USA; 2 Oral and Maxillofacial Surgery, University of Florida College of Medicine – Jacksonville, Jacksonville, USA

**Keywords:** granular cell tumour, maxilla mass, oral cavity tumors and cyst, pathology, soft tissue tumours

## Abstract

Non-neural granular cell tumors (NNGCTs) represent a rare variant of granular cell tumors that lack S100 expression and do not demonstrate nerve-sheath differentiation, distinguishing them from conventional granular cell tumors (CGCTs), which are typically S100-positive and of Schwann cell origin. This report describes the case of a 46-year-old male presenting with a soft tissue mass arising from the right maxillary alveolar ridge. A hemimaxillectomy was performed, and the mass was sectioned to reveal light tan fibrous tissue grossly involving the underlying bone. The mass was composed of large neoplastic polygonal cells with granular cytoplasm that showed mild nuclear atypia and a low mitotic count. Immunohistochemical staining showed that the cells were positive for CD68 and CD45 expression but were negative for S100. The final diagnosis was reported as NNGCT. The prognosis, genetic alterations, and cellular origins of these tumors remain poorly understood. The findings included in this report will hopefully inform future investigations into the nature of NNGCTs and ultimately lead to improved treatment outcomes.

## Introduction

Conventional granular cell tumors (CGCTs) are a subset of soft tissue tumors composed of large polygonal-shaped cells with eosinophilic, granular cytoplasm. Non-neural granular cell tumors (NNGCTs), first described by Leboit et al. and originally given the name primitive polypoid granular-cell tumors [1], are differentiated from CGCTs in that they do not show Schwannian differentiation and are negative for expression of S100. Despite histologic features typically worrying for malignancy, such as a high number of mitotic figures and a high degree of pleomorphism, the large majority of NNGCTs are benign [2,3]. Herein, we describe a case of a patient who presented with a large, slow-growing solid mass in his oral cavity. This report details the process by which a diagnosis of NNGCT with bone invasion was reached; however, the clinical significance remains uncertain given the limited literature regarding this entity.

## Case presentation

**Figure 1:**
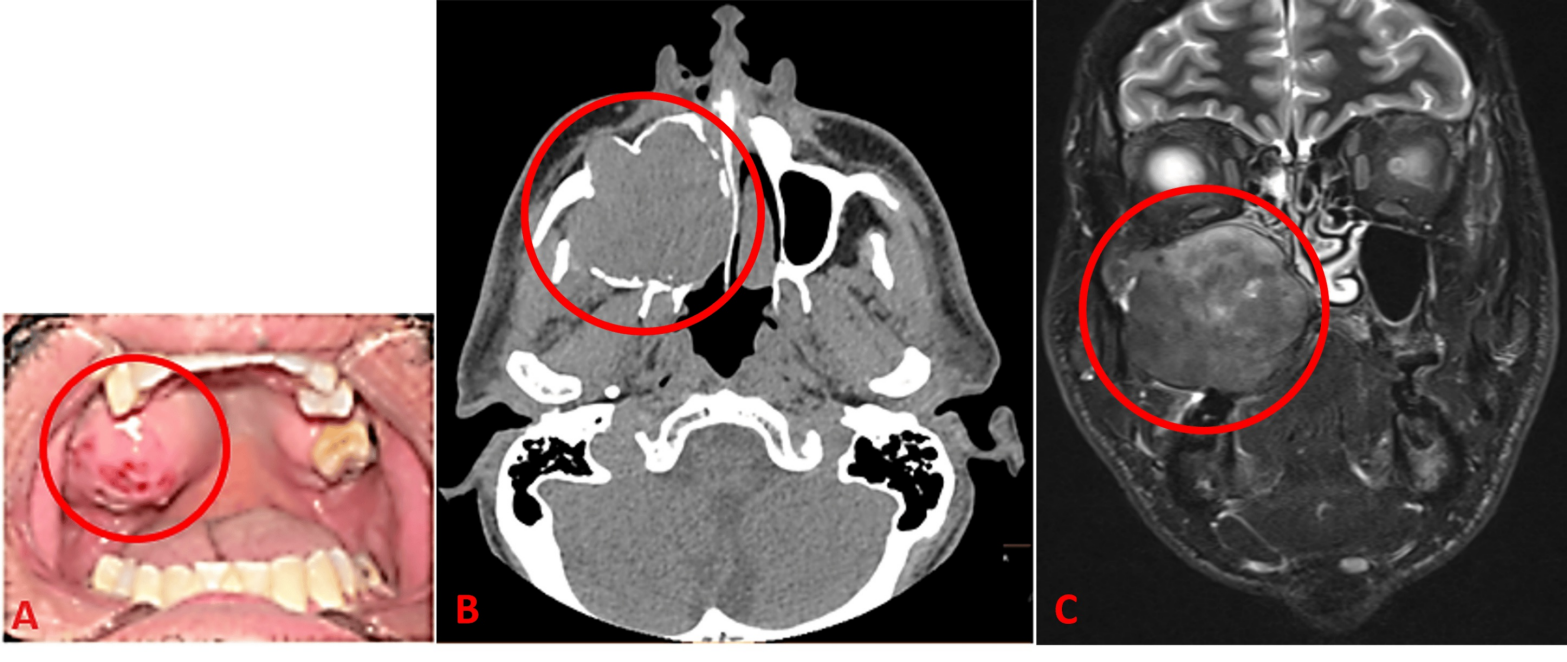
Images of the mass The patient’s oral cavity and maxillary mass (A; mass in red circle). CT (B) and MRI (C) images showing a mass with bone invasion of the maxilla and walls of the maxillary sinus (red circles).

A 46-year-old male with past medical history notable only for a >28 pack-year smoking history initially presented with a painless, firm, roughly spherical mass arising from the right maxillary alveolar ridge identified during a dental procedure. He was referred to oral and maxillofacial surgery for evaluation and biopsy. Initial biopsy was returned as leiomyoma at an outside institution. A follow-up biopsy performed in the clinic returned as granular cell neoplasm. The patient reported that the mass had grown slowly over three months leading up to the dental procedure, but that the mass had been mostly stable in size since his presentation to oral and maxillofacial service (Figure 1A). The patient denied any visual changes, neurosensory disturbances, pain, drainage, or facial swelling. A computerized tomography (CT) scan showed a stable expansile soft tissue mass approximately 5.3 × 4.9 × 4.6 cm in size arising from the right maxillary alveolar ridge with diffuse thinning/erosion of the right maxillary sinus walls (Figure 1B). A subsequent magnetic resonance image (MRI) further demonstrated an intensely enhancing, homogeneous mass involving the right maxillary alveolar ridge and right maxillary sinus and obstructing the right nasolacrimal duct (Figure 1C). No significant lymphadenopathy or distant metastases were identified.

**Figure 2:**
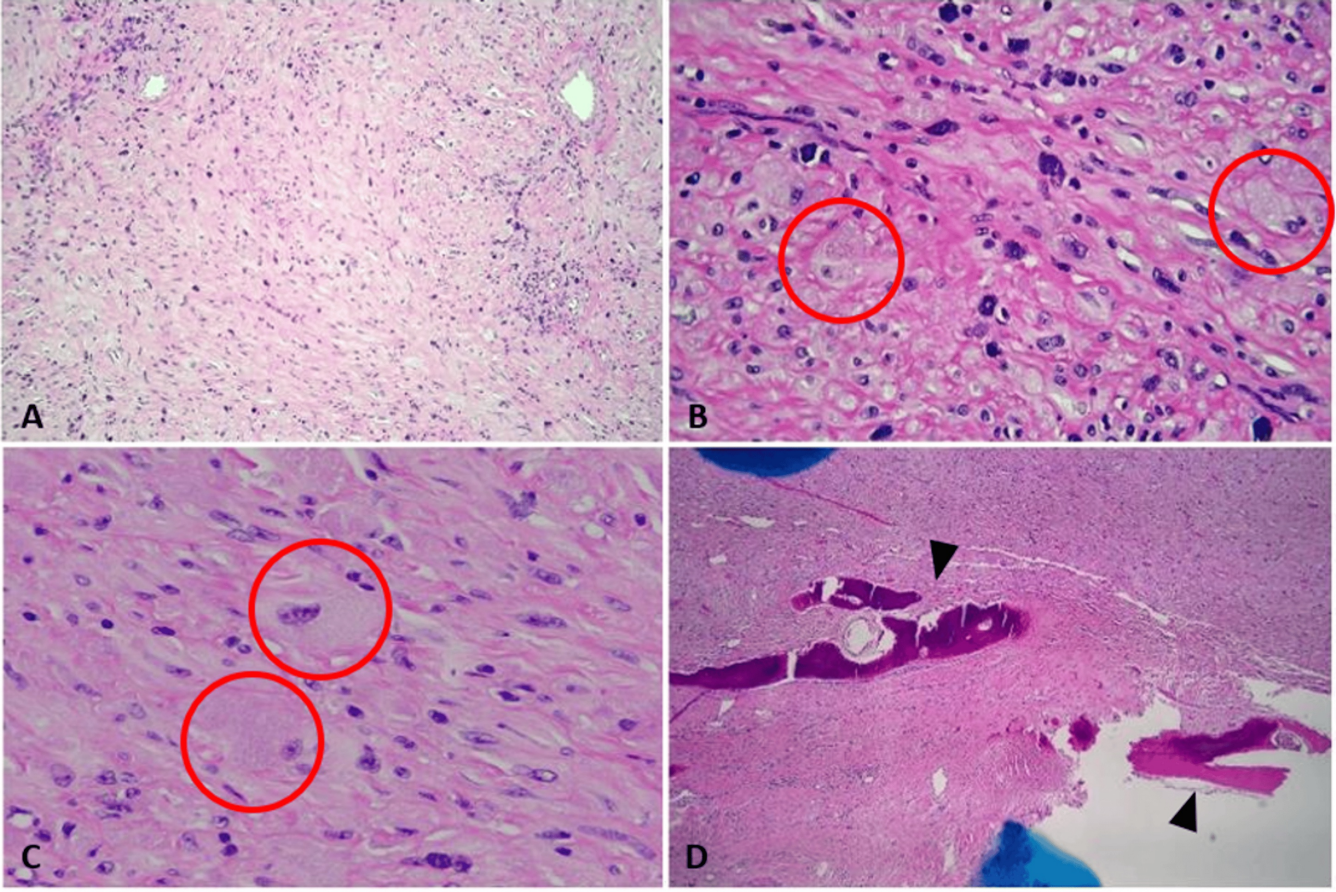
Microscopic morphology Hematoxylin and eosin (H&E) showing tumor and cellular morphology at 10X (A), 40X (B), and 60X (C) consisting of polygonal cells with granular eosinophilic cytoplasm (red circles) and prominent plasma cell component. Invasion of maxillary bone demonstrated at 5X (D, black arrowheads).

A hemi-maxillectomy was performed, and the mass, measuring 5.6 cm in greatest dimension, with a fragment of attached bone on the medial surface and attached teeth on the lateral surface, was received by the pathology department. The specimen was serially sectioned to reveal light tan fibrous tissue originating grossly from the alveolar ridge of the maxilla with involvement of the underlying bone. Hematoxylin and eosin (H&E)-stained slides showed neoplastic large polygonal cells with granular cytoplasm intermingled with spindle cells that showed mild nuclear atypia in a loose fibrocollagenous stroma and a prominent mast cell component (Figures 2A, 2B, 2C). Mitotic count was low, approximately 1 in 10 high-power fields (HPF). No necrosis was identified. The tumor appeared to invade through the maxillary bone (Figure 2D), but no marked atypia was identified. Margins of the hemi-maxillectomy were negative for tumor.

**Figure 3:**
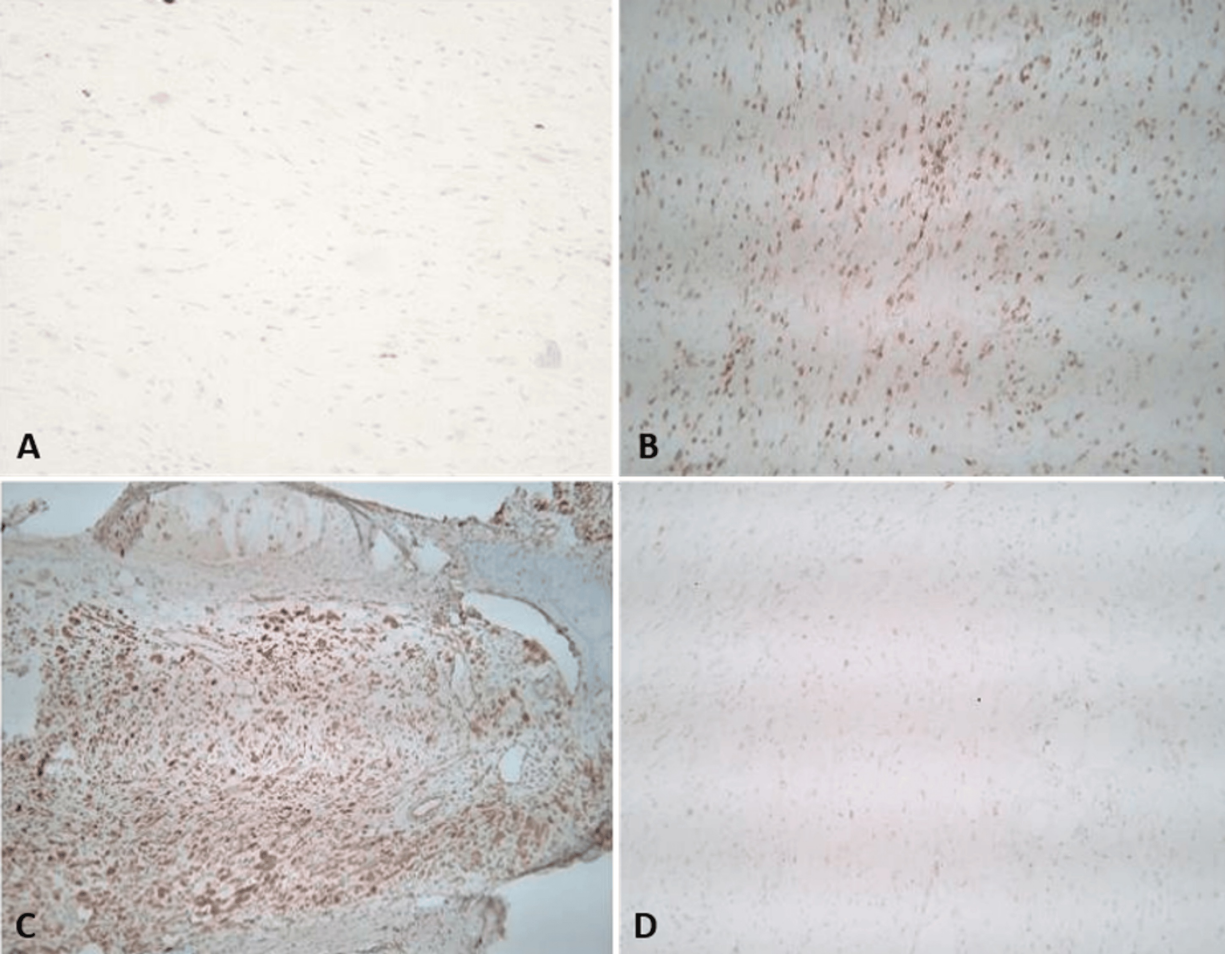
Immunohistochemistry Immunohistochemical stains showing S100 negative (A), CD45 positive (B), CD68 positive (C), and Inhibin patchy positive (D).

Based on gross and microscopic morphology, the final diagnosis of NNGCT was made after excluding other oral cavity tumors such as squamous cell carcinoma and ameloblastoma, CGCT, atypical leiomyoma, atypical fibroxanthoma, dermatofibroma, rhabdomyoma, ganglioneuroma, and melanoma. Immunohistochemical staining (Figure 3A) showed that the neoplastic cells were negative for S100, CD56, SOX10, HMB-45, myogenin, calretinin, AE1/AE3, desmin, Melan-A, SMA, and CD34, showing only background nonspecific staining. These results allowed for the ruling out of CGCT (S100 and SOX10 positive), leiomyoma (SMA and desmin positive), rhabdomyoma (myogenin and desmin positive), ganglioneuroma (S100 positive), and melanoma (HMB-45 and Melan-A positive). The neoplastic cells were positive for CD45 and CD68 (Figures 3B, 3C) and showed patchy positivity for inhibin (Figure 3D), while the Ki-67 proliferative index was approximately 5-10%, indicating a relatively slow-growing and less aggressive tumor. These results also supported the final diagnosis of NNGCT.

The patient continued to follow up with oral and maxillofacial surgery for monitoring and maxillary reconstruction. No chemotherapy or other medication to prevent possible recurrence was prescribed. Though the patient later required additional oral surgeries, including the extraction of multiple decaying teeth, no tumor recurrence has been reported at six months post-surgery.

## Discussion

CGCT is a rare entity composed of cells with features most consistent with Schwann cell (nerve sheath) differentiation. These cells are characterized by abundant cytoplasmic granules, believed to be increased lysosomes [4,5]. The cells also show strong and diffuse immunoreactivity for S100 protein, p75/NGFR, and neuron-specific enolase, supporting a neuronal origin [4,5]. NNGCTs demonstrate histomorphological features similar to CGCTs but lack evidence of neural differentiation. Immunohistochemically, NNGCTs are positive for markers such as CD68, NKI/C3 (CD63), vimentin, and sometimes factor XIIIa or cyclin D1, but negative for S100, neuron-specific enolase, and other neural, melanocytic, or myoid markers (Table 1) [6,7]. The histogenesis of NNGCTs remains uncertain. A possible relationship to mesenchymal stem cells or hair follicle structures has been suggested [7,8], with some molecular studies identifying recurrent ALK gene fusions in a subset of cases [9], further differentiating NNGCTs as a distinct entity from CGCTs.

**Table 1:** Non-neural granular cell tumor versus conventional granular cell tumor Comparison of immuno-expression between non-neural granular cell tumors (NNGCTs) and conventional granular cell tumors (CGCT)

	S100/NSE	Cyclin D1	CD68	ALK	CD63 (NKI/C3)
CGCT	Positive [9,10]	Negative [10]	Positive [10]	Negative [9]	Negative [9]
NNGCT	Negative [9,10]	Positive [10]	Positive [10]	Positive in subset [9]	Positive [9]

Clinically, NNGCTs usually present as painless, polypoid or nodular lesions, particularly on the skin of young to middle-aged adults, with a slight female predominance, and usually present as a firm, nodular mass arising from the dermis of the trunk, head and neck, or upper limbs [10-12]. Histologically, they are composed of large polygonal or spindle-shaped cells with abundant granular eosinophilic cytoplasm that may show greater nuclear atypia and increased mitotic activity in comparison to the CGCTs, with rare instances of regional lymph node metastasis reported [13]. Granular cell tumors are classified using the Fanburg-Smith criteria as benign, atypical, or malignant. The six criteria include high nuclear cytoplasmic ratio, pleomorphism, vesicular nuclei with prominent nucleoli, spindling, necrosis, and mitoses [5]. Metastatic disease remains the definitive evidence of malignancy [5]. It has previously been reported that greater nuclear atypia and increased mitotic activity are expected in NNGCTs compared to CGCTs. However, the Fanburg-Smith criteria are still referenced in the context of NNGCTs [9]. In the case presented in this report, no significant atypia, necrosis, or high numbers of mitoses were identified.

The case presented in this report featured an NNGCT originating in the oral cavity from the alveolar ridge of the maxilla with bone and sinus invasion (Table 2). In previously published reports, NNGCTs are typically described as poorly circumscribed masses, the majority of them having presented mainly in the extremities, with infiltration to the surrounding tissue [2,7]. However, to the best of our knowledge, no bone invasion has previously been reported in an NNGCT. Bone invasion can rarely present in CGCTs [14], but it is not considered a definitive criterion for malignancy, and the clinical significance remains uncertain [15]. Although NNGCTs exhibit benign behavior and are treated definitively with surgical resection in the majority of reported cases [2], very rare cases of metastases and lymph node involvement have been reported [16]. Given the lack of medical literature surrounding NNGCT and bone invasion, close follow-up for recurrence and possible metastasis may be indicated. Despite the novel and/or atypical features demonstrated by the tumor described by this report, the morphologic and immunohistochemical findings support the final diagnosis.

**Table 2:** Summary of results

Element of the case	Results
Patient details	46 years old, male, > 28 pack-year tobacco smoking history, no history of prior malignancy
Tumor size	5.6 cm
Clinical features	Slow growing, painless, no drainage/discharge, no neurosensory symptoms
Gross morphology	Round, solid, rubbery, light tan, fibrous tissue
Microscopic morphology	Large polygonal cells with granular cytoplasm, intermingled spindle cells with mild nuclear atypia, loose fibrocollagenous stroma, prominent mast cells
Positive immunostains	CD45, CD68, and inhibin (patchy)
Negative immunostains	S100, CD56, SOX10, HMB-45, myogenin, calretinin, AE1/AE3, desmin, Melan-A, SMA, and CD34
Ki-67 index	5-10%

## Conclusions

This report describes the case of a solid mass arising from the maxilla of a 46-year-old male patient with no previous history of neoplastic disease. Features such as eosinophilic, granular cytoplasm, as well as negative immunostaining for S100, assisted in reaching a final diagnosis of NNGCT. The prognosis, genetic alterations, and cellular origins of NNGCTs remain poorly understood, and the literature describing the entity is relatively lacking. While it is difficult to extrapolate about NNGCTs based on the results of a single case, the findings included in this report will hopefully inform future investigations into the nature of NNGCTs and ultimately lead to improved treatment outcomes.
